# Rapid isolation and quantification of extracellular vesicles from suspension‐adapted human embryonic kidney cells using capillary‐channeled polymer fiber spin‐down tips

**DOI:** 10.1002/elps.202200149

**Published:** 2022-09-04

**Authors:** Kaylan K. Jackson, R. Kenneth Marcus

**Affiliations:** ^1^ Department of Chemistry Clemson University Clemson South Carolina USA

**Keywords:** capillary‐channeled polymer, extracellular vesicles, HEK293 cell culture, solid‐phase extraction, therapeutic vectors

## Abstract

Exosomes, a subset of extracellular vesicles (EVs, 30–200‐nm diameter), serve as biomolecular snapshots of their cell of origin and vehicles for intercellular communication, playing roles in biological processes, including homeostasis maintenance and immune modulation. The large‐scale processing of exosomes for use as therapeutic vectors has been proposed, but these applications are limited by impure, low‐yield recoveries from cell culture milieu (CCM). Current isolation methods are also limited by tedious and laborious workflows, especially toward an isolation of EVs from CCM for therapeutic applications. Employed is a rapid (<10 min) EV isolation method on a capillary‐channeled polymer fiber spin‐down tip format. EVs are isolated from the CCM of suspension‐adapted human embryonic kidney cells (HEK293), one of the candidate cell lines for commercial EV production. This batch solid‐phase extraction technique allows 10^12^ EVs to be obtained from only 100‐µl aliquots of milieu, processed using a benchtop centrifuge. The tip‐isolated EVs were characterized using transmission electron microscopy, multi‐angle light scattering, absorbance quantification, an enzyme‐linked immunosorbent assay to tetraspanin marker proteins, and a protein purity assay. It is believed that the demonstrated approach has immediate relevance in research and analytical laboratories, with opportunities for production‐level scale‐up projected.

**Abbreviations**
CCM: cell culture milieu
C‐CP: capillary‐channeled polymer
EV: extracellular vesicle
HIC: hydrophobic interaction chromatography
NTA: nanoparticle tracking analysis
PET: polyethylene terephthalate

## 1 INTRODUCTION

As primary vehicles of intercellular communication, nanometer‐scale extracellular vesicles (EVs) allow for bioactive cargos to be transferred between cells in close and far proximity, even crossing barriers of bodily systems [1]. EVs are secreted by all living cells and are composed of a phospholipid bilayer membrane, and contain lipid, protein, and genetic (DNA, mRNA, miRNA) cargos from the cell of origin [1, 2, 3, 4]. Overall, EV populations are heterogeneous in size (30–4000 nm), composition, and function, reflecting the original microenvironment from which they were secreted and their mode of creation [5]. Depending on the state of the origin cell, secreted EVs can contribute to either the maintenance of normal/healthy physiology or the progression of disease [6, 7, 8, 9]. The abundance of EVs in excreted biofluids (i.e., urine, saliva, blood) has made them ideal targets for liquid biopsies, whereas cell culture milieu (CCM) are means of EV production for therapeutic vector applications [10, 11].

Limiting EV applications are the lack of understanding of EV fundamentals, the inability to well characterize EV subtypes, and potentially most limiting: the absence of scalable methods to isolate pure, populated collections of EVs and quantify them efficiently [12, 13, 14]. The three main subclasses of EVs are (1) microvesicles (MVs), which are shed from the cell membrane of living cells, ranging from 50 to 1000 nm in size [1, 15]; (2) apoptotic bodies of 50–4000‐nm diameter, which are stochastically released from dying cells [16, 17]; and (3) exosomes, smaller EVs (sEVs) of roughly 30–200‐nm diameter, uniquely created through the multivesicular body–mediated endosomal pathway and released via exocytosis [10, 11, 12]. Of the EV subtypes, exosomes are considered the “main mediators” of cellular communication to affect functional changes in the recipient cell [18]. However, the effective isolation of exosomes from other EV types is particularly challenging, so the assignment of exosome‐specific activities to functional responses has been impeded [19]. Moreover, the overlapping of the exosome and MV size ranges and similarities in composition and morphology have led to collections of vesicles in the sEV size range (50–200 nm) to be generically referred to as EVs [12, 20].

Because EVs are cell secretion products, the production of concentrated pools of EVs depends upon the ability to provide large quantities of cells in a way that does not cause alterations in the cellular phenotype (thereby, EV cargos) [13, 21, 22]. Of the many cell types, human embryonic kidney (HEK293) cells are prime candidates for the scalable production of EVs, with previous successes in the production of recombinant proteins, monoclonal antibodies (mAbs), and adeno‐associated virus vectors for biotherapeutics [23, 24, 25, 26, 27]. Previous works have demonstrated that after the harvest of the EVs from HEK293 cells, they can be bioengineered to contain specific gene, drug, or protein contents for therapeutic applications ranging from opioid addiction [28] to cancer [29]. In all, HEK293‐derived EVs hold the potential to provide a means of delivering powerful drug and gene therapies in a way that is practical in terms of cost and scalability.

In order to affect better EV production, several HEK293 cell lines, such as the HEK293T/17 SF cell line from American Type Culture Collection (ATCC), have been conditioned for growth in suspension serum‐free cell culture environments [30, 31]. Although the challenges of future production‐scale isolation/purification of EVs are immense, the inability to perform high‐throughput, high‐purity separations on clinical/research scales of single milliliters has prevented better fundamental research. (The same can be said for potential clinical diagnostic applications of EVs.) There is much to be learned to affect the better production of targeted EV populations, and so there are gains to be made in terms of fundamental biochemistry if better analytical strategies could be implemented. Along the same lines, suitable analytical‐scale methods would take a position as part of the process monitoring toolbox in EV production. Taken a step further, the demonstration of strategies for high fidelity isolation/purification at analytical scales could yield platforms suitable for implementation on the preparative scale.

Marcus and colleagues have developed a hydrophobicity‐based EV isolation method employing capillary‐channeled polymer (C‐CP) fiber stationary phases to address the shortcomings of the currently available EV isolation methods [32, 33, 34, 35, 36, 37, 38, 39, 40]. These C‐CP fiber phases have been employed in highly efficient EV isolations via high‐performance liquid chromatography (HPLC) [32–34, 38, 39] and solid‐phase extraction (SPE) tip [35–37, 40] formats, concentrating on what would be called analytical‐scale processing. In both cases, the isolation of EVs is driven by an organic modifier‐assisted hydrophobic interaction chromatography (HIC) solvent system, where EVs have been obtained from several complex biofluids, including urine, saliva, blood serum, cervical mucus, and CCM from *Dictyostelium discoideum* cell culture [32, 35, 39]. In all cases, high concentrations of EVs (up to 7 × 10^12^ EVs ml^−1^) have been obtained from sub‐milliliter initial sample volumes, with over 95% removal of contaminating proteins and lipoproteins as confirmed by mass spectrometric (MS) proteomics analysis [34, 36]. Thus, the method allows collections of EVs fit for fundamental research and clinical assays, as well as potential use for production system process monitoring.

In previous C‐CP‐based HIC isolations of EVs, acetonitrile (ACN) and glycerol solvent additives were utilized to aid the elution of the vesicles from the fiber surface [32, 39]. The ACN solvent additive was proven most compatible for EVs subsequently analyzed by MS, RNA sequencing, and immune characterization approaches [34, 38]. Nevertheless, the high concentrations of ACN are not ideal for the long‐term stability of EVs, though most of the latent ACN can be removed using a simple off‐gassing process under a low vacuum. Alternatively, a glycerol solvent modifier was introduced for use in the case when the long‐term structural preservation of the EVs was the end goal [39, 40]. Though the glycerol solvent does provide cryopreservative properties, the high viscosity of the solvent can prevent the accurate assessment of the vesicles during proteomic analysis, immunoassays, and flow cytometry assays [41, 42]. Though some latent glycerol can be removed via a post‐processing ultrafiltration step, there can still be some interference with downstream analyses due to remnant glycerol content blocking access to surface proteins, and so on. Overall, though the ACN and glycerol HIC solvent additives were able to provide high concentrations of pure EVs, both solvent types can limit the characterization and utilization of recovered EVs [40]. To affect EV separations without the required post‐isolation solvent removal steps, where a portion of the recovered EVs may be lost due to transfer, the identification of alternative elution solvents is of interest. As an alternative to the formerly used solvent additives, a Tween‐20 EV elution solvent was considered, as Tween‐20‐based solvents are common to many standard immunoassays and EV analysis (i.e., Spectradyne) protocols [43, 44]. Studies have also suggested that the exosome EV‐subtype is resistant to detergent activity, and the morphology of the exosomes is unaffected by low concentrations of detergents (including Tween‐20) [44, 45]. As the HIC C‐CP tip isolation process is driven by a high‐to‐low salt solvent transition, an aqueous Tween‐20 solution could be utilized as an organic modifier in the isolation workflow for the final elution of EVs. As with the other elution matrices, there are likely applications where Tween is not acceptable, such as MS proteomics workflows.

In this report, a C‐CP‐based HIC isolation of EVs from suspension‐adapted human embryonic kidney cells (HEK293T/17 SF) grown in a serum‐free environment is performed. Aliquots of CCM from varying time points in cell growth were collected and processed using the HIC C‐CP spin‐down tip method with the Tween‐20‐based EV elution buffer. A comprehensive suite of characterization methods has been employed to follow the recovery characteristics of the EVs. Transmission electron microscopy (TEM) was used to verify the size, shape, and structural integrity of the EVs recovered using the C‐CP tip method. A simple, flow‐through multi‐angle‐light scattering (MALS) detection apparatus was used to determine the size of the recovered EVs. The method of standard addition using absorbance (scattering) detection was used for the efficient quantification of EVs. A Bradford assay was used to monitor the concentration of protein eluted at each step in the isolation process and assess the purity of the vesicles based on the removal of host cell proteins. Finally, an indirect enzyme‐linked immunosorbent assay (ELISA) using antibodies to the CD9 and CD81 exosomal surface marker proteins was used to confirm the presence and bioactivity of the collected EVs. In summary, the C‐CP tip isolation method employing the Tween‐20 solvent additive was able to rapidly provide high concentrations of high‐purity EVs while being compatible with every characterization method utilized. It is believed that the approach demonstrated here has immediate relevance in research and analytical laboratories, with opportunities for production‐level scale‐up projected.

## 2 MATERIALS AND METHODS

### 2.1 Chemicals, solvents, and antibodies

Deionized water (DI‐H_2_O, 18.2 MΩ cm) was obtained from a Milli‐Q water purification system (Millipore Sigma, Merck, Darmstadt, Germany). Ammonium sulfate and Tween‐20 were purchased from VWR (Solon, OH, USA). Phosphate‐buffered saline (PBS, pH = 7.4) and bovine serum albumin (BSA) were purchased from Thermo Fisher Scientific (Waltham, MA, USA). Paraformaldehyde and formvar/carbon 200‐mesh copper grids were obtained from Electron Microscopy Science (Hatfield, PA). Polyclonal rabbit anti‐CD9 and CD81 primary antibodies and a goat anti‐rabbit HRP‐conjugated secondary antibody were obtained from System Biosciences (SBI, Palo Alto, CA). The Pierce Coomassie Plus (Bradford) Assay Reagent was purchased from Thermo Fisher Scientific (Waltham, MA, USA).

### 2.2 Commercial exosomes

Lyophilized exosomes of 3.6 × 10^11^ particles ml^−1^ concentration from the cell culture media of human embryonic kidney (HEK293) cells were obtained from HansaBioMed (Tallinn, Estonia). Per the manufacturer's instructions, the 100 µg of lyophilized exosomes were reconstituted in 100 µl of Milli‐Q water before being applied to future characterization and quantification approaches. Though the commercial exosome material provides a point of reference for the quantification of EVs, this exosome stock is not a certified reference material; that is, no quantitative/qualitative values to reflect the purity and exclusivity of the exosome stock are supplied. Indeed, no such materials are commercially available. Despite these limitations, the commercially obtained EV “standards” do serve as an EV stock of known concentration, providing a basis for EV quantification.

### 2.3 HEK293T/17 SF cell culture

A human embryonic kidney (HEK293T/17 SF) cell line, adapted for serum‐free suspension cell culture conditions, was obtained from ATCC (Manassas, VA, USA). The HEK293T/17 SF cell line was cultured in BalanCD HEK293 cell culture media (Irvine Scientific, Santa Ana, CA, USA), supplemented with 8‐mM l‐glutamine and 10 µm ml^−1^ of insulin–transferrin–selenium (ITS, Corning, Corning, NY, USA) on a 37°C shaking incubator (160 rpm) with 5% CO_2_. A Vi‐CELL XR Cell Viability Analyzer (Beckman Coulter, Brea, CA, USA) was used to determine the concentration and viability of the cell line, employing the trypan blue dye exclusion method [46]. It must be noted that the conditions employed here are considered to be typical and not intended to represent the state of the art in HEK culture technology.

### 2.4 C‐CP SPE tip assembly

C‐CP fiber micropipette tips were prepared through the previously described process [35–37, 40]. The fibers were formed via melt‐extrusion from bulk polyester (polyethylene terephthalate, PET) in the Clemson University School of Materials Science, having the form of an eight‐pronged shape of ∼24 × 38‐µm cross section. To create the C‐CP tips, eight rotations of the PET fiber bundles (57 fibers per bundle, 456 polymer fibers total) were collinearly aligned, preshrunk with boiling water, washed with ACN, water, then ACN to remove any lingering static coatings, and pulled through a 30‐cm‐long segment of fluorinated ethylene propylene tubing of 0.8‐mm inner diameter. The fibers colinearly packed inside of the column were cut to create 1‐cm fiber‐packed tips, with an additional 0.5 cm of empty tubing allowing the columns to be attached to a 200‐µl low‐retention micropipette tip (SureOne Micropoint Pipette Tips, Universal Fit, Non‐Filtered, Fisherbrand, Pittsburgh, PA), which was held in place with a small amount of liquid adhesive. The C‐CP‐modified micropipette tip was then placed inside a 1‐ml micropipette for structural support and secured inside a 15‐ml conical tube using a customized adapter cap to hold the C‐CP tip.

### 2.5 EV isolations using the HIC elution protocol

An HIC solvent system was used with the C‐CP tips to isolate the EVs from the HEK293T/17 SF EVs cell culture media. For this, 200 µl of the cell culture supernatant was filtered using a 0.22‐µm PES filter, then mixed with an equal part of ammonium sulfate (2‐M final concentration), with the 400‐µl mixture applied to the C‐CP tip. The entire tip apparatus was placed in the turret of a tabletop centrifuge (Symphony 4417, VWR) and spun down at 300 × *g* (rcf) for 1 min. The higher hydrophobicity species (i.e., proteins and EVs) are captured on the fiber tip surface during the initial spin‐down step, whereas the small ionic/hydrophilic sample components (i.e., salts, sugars, and amino acids) pass unretained. To remove the free host cell protein and lipoprotein contaminants, 200 µl of the protein elution buffer containing 25% ACN with 1‐M ammonium sulfate was loaded into the C‐CP tip reservoir and spun down at 300 × *g* for 1 min. This protein elution step was repeated to ensure that all contaminant protein/lipoprotein species had been removed. Finally, to release the now‐purified EVs from the fiber tip surface, 100 µl of an EV elution buffer consisting of 1% Tween‐20 in PBS was applied to the C‐CP tip and centrifuged at 300 × *g* for 1 min.

### 2.6 Transmission electron microscopy (TEM)

TEM imaging, performed using a Hitachi HR7830, was used to provide the physical identification of cup‐shaped EVs after processing the cell culture media collections via the C‐CP tips. In preparation for TEM imaging, 7 µl of each HEK‐EV recovery was placed on an EM‐grade copper/formvar grid and incubated at room temperature for 20 min. The excess sample liquid was then removed using a paper towel, and the EVs on the grids were immediately fixed using 2% paraformaldehyde (RT, 5 min). After fixation, the excess paraformaldehyde was removed from the grids using a paper towel before gently washing them with water for 5 min. Next, the EVs immobilized on the grids were stained using a filtered 1% uranyl acetate solution (RT, 1 min), the excess staining solution was removed, and the prepared grids were again washed with water. Finally, the prepared TEM grids were allowed to dry in a cell culture dish for 30 min in a desiccator at room temperature before imaging. The size of the vesicles visualized in the TEM micrographs was determined using ImageJ.

### 2.7 Absorbance quantification using the method of standard addition

This laboratory has previously reported the use of standalone UV–Vis spectrometers to determine EV concentrations following spin‐down tip processing, employing standard response curves, and the method of standard addition [35, 36, 40]. In this work, the EVs from HEK293T/17 cell culture media were quantified via standard additions as it shows greater precision for complex matrices. Here, recovered EVs are spiked once, twice, and three times with the commercial exosome standards (3.6 × 10^11^ particles ml^−1^) derived from HEK293 cells, using the absorbance at 203 nm using a NanoVue Plus UV–Vis spectrophotometer (GE Healthcare, Chicago, IL, USA). Though this exosome standard stock is not a standardized reference material, a general approximation of EV quantification can be obtained.

### 2.8 Size determinations using multi‐angle light scattering (MALS) detection

A DAWN MALS detector (Wyatt Technology, Goleta, CA), controlled using the ASTRA software, was used for the size determination of the recovered HEK‐EVs. After isolating the EVs from the bulk cell culture media, 20 µl of each eluate was injected and transferred to the MALS detector at 0.5 ml min^−1^ using a Dionex Ultimate 3000 HPLC system (LPG‐3400SD quaternary pump and MWD‐3000 UV–Vis absorbance detector, Thermo Fisher Scientific, Sunnyvale, CA, USA) controlled by the Chromeleon 7 software. The MALS‐determined RMS radii were then multiplied by 2 to represent the approximate diameter/size of the vesicles. Throughout the MALS analysis, the refractive index was set to that of 1% Tween in PBS at 22°C, 1.3363, which was determined experimentally using a Reichert AR7 Series Automatic Refractometer. Three replicate measurements were collected for each sample in 60‐s increments.

### 2.9 Isolate purity verification by Bradford assay

A critical EV purity metric has become the number of EVs with respect to the total protein content in the isolates [12, 47]. A standard Bradford assay was used to determine the total protein concentration of each CCM sampling and the protein content of the respective C‐CP tip elution fractions (protein and EV). Here, it is important to emphasize that even in the case of pure EVs, there will be some positive response toward the Bradford assay due to the proteins incorporated in the vesicle walls. For total protein determinations, 25 µl of each sample was combined with 250 µl of Bradford reagent in a 96 cell well plate and incubated on a shaker at room temperature for 20 min before the detection of absorbance response at 595 nm using the Synergy H1 Hybrid Plate Reader (BioTek, Winooski, VT). The sample absorbance responses were compared to a BSA standard curve of linear response to determine the total protein concentration. All samples and standards were applied to the 96 cell well plate in triplicate, and triplicate absorbance measurements were performed.

### 2.10 EV identity confirmation using an enzyme‐linked immunosorbent assay (ELISA)

To verify the presence of bioactive EVs (based on tetraspanin protein expression) after the C‐CP tip isolation process, an indirect ELISA employing antibodies to the CD9 and CD81 tetraspanin proteins was used. For this, 50 µl of each C‐CP tip eluate was applied to the ELISA 96 cell well plate with equal volumes of ELISA coating buffer (0.05‐M carbonate–bicarbonate in PBS) and allowed to incubate overnight at 4°C. Each sample was applied in triplicate, along with triplicate applications of an exosome standard positive control and negative controls of PBS and the respective protein and EV elution buffers. Following this sample incubation, each well was washed with 200 µl of sterile PBS (six buffer changes, 30 min total) before a 5% BSA blocking solution was applied and allowed to incubate on a shaker at room temperature for 30 min. A volume of 200 µl of the anti‐CD9 and anti‐CD81 antibody solutions of 1‐µg ml^−1^ concentration was added to each sample well and allowed to incubate overnight on a shaker at 4°C. Following incubation, the washing and blocking steps were repeated as done previously. A volume of 200 µl of the goat anti‐rabbit HRP‐conjugated secondary antibody (1 µg ml^−1^) was applied to each sample well and allowed to incubate at room temperature for 2 h. Here again, the cell well plate was washed using 200 µl of PBS per well (six buffer changes, 30 min total) before applying 50 µl of the 1‐Step Ultra TMB‐ELISA Substrate Solution. The colorimetric ELISA reagent was allowed to incubate for 30 min at room temperature before the absorbance response was measured at 562 nm using the Synergy H1 Hybrid Plate Reader.

## 3 RESULTS AND DISCUSSION

### 3.1 Cell concentration and viability as a function of culture time

Previous reports have shown that changes in the concentration of EVs can be used to assess the health of a cell line [48], with the upregulated release of EVs being attributed to environments or situations contributing to cell stress, and a decrease in release of EVs as being a response to nutrient depletion. Because of these sorts of relationships, scientists have suggested that a simple EV quantification/characterization method could provide insight into the productivity of a cell line, which could be particularly useful in large‐scale bioreactor applications for therapeutics [13, 49]. Herein lies the potential use of a rapid EV characterization tool as in a process monitoring mode. Intuitively, essential factors to assess are the viable cell concentration and the concentration of cell secretion products. The purpose of this study is to potentially characterize the state of an HEK293 cell culture based on EV release at various time points in the cell culture process using the C‐CP spin‐down tip method.

**FIGURE 1:**
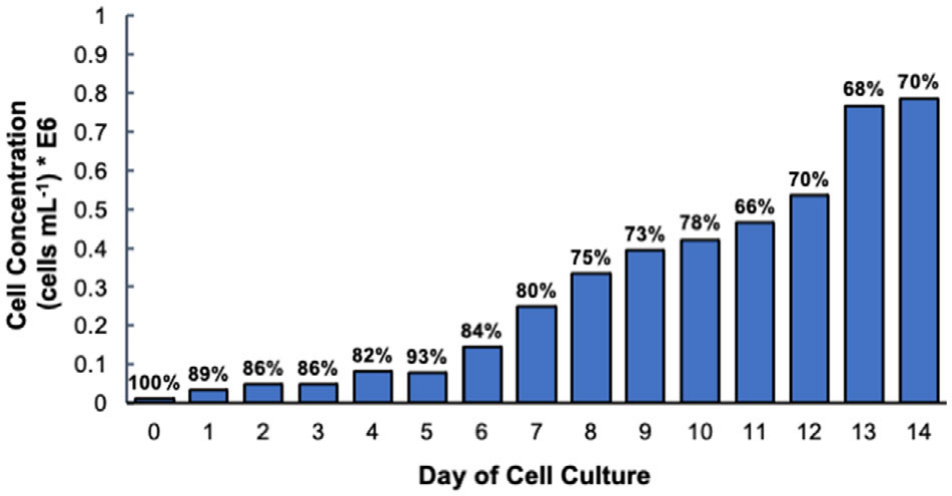
Concentration of HEK293 cells in native cell culture milieu (CCM) supernatant with the percentage viability on each day of cell culture as determined using the Vi‐Cell XR instrument via trypan blue cell exclusion assay

Shown in Figure 1 are the growth characteristics of the HEK293T/17 SF cells as a function of culture time. An exponential growth phase, which is a characteristic of healthy HEK293 cell growth [50, 51, 52, 53], is observed (*R* ^2^ = 0.9236), with 66%–100% of the cells collected at each time point determined as viable based on the trypan blue dye exclusion method. Typically, a collection of cells with a percentage viability of greater than 80% is considered to be a “healthy” culture [54, 55]. In this case, the cells on days 0–7 of cell culture fall within the healthy cell viability range but decreases below 80% viability on days 8–14 of culture. To assess the EV release throughout the 14‐day time window, collections of CCM from each time point were processed using the C‐CP tip isolation method to provide concentrated, representative EV populations for further characterization.

### 3.2 Structural verification using transmission electron microscopy (TEM)

**FIGURE 2:**
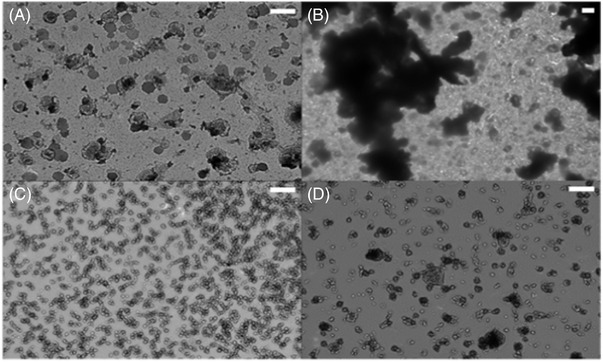
Transmission electron micrographs of eluates from each step in the hydrophobic interaction chromatography (HIC) capillary‐channeled polymer (C‐CP) tip extracellular vesicle (EV) process. Representative micrographs from the (A) native HEK293 cell culture milieu (CCM) supernatant, (B and C) exposure to first and second protein elution buffers, and (D) the EV elution buffer. The transmission electron microscopy (TEM) images were taken using the Hitachi HR7830, scale bar = 200 nm.

Though many EV characterization approaches are available, TEM remains the “gold standard” technique to visually confirm EV characteristics, such as size and the cup/spherical EV shape [19]. TEM was used as a benchmarking approach to verify that the EVs were present in the original CCM sample, and that their physical characteristics were retained during the subsequent isolation of EVs from CCM using the C‐CP tip method with the 1% Tween EV elution buffer. Representative TEM micrographs for the HEK293 CCM starting material (Figure 2A) and the eluate from each C‐CP tip isolation step (Figure 2B–D) are presented in Figure 2 (scale bar = 200 nm). Indeed, in Figure 2A, EVs of 110‐nm average diameter are observed in the CCM stock, with the characteristic spherical and dimpled shapes. Some EV aggregates and potential proteinaceous contaminants are also observed in the field of view, with some vesicles being >200 or <50 nm in diameter.

After applying the CCM sample to the C‐CP tip and proceeding with the first protein elution step, matrix‐originating components, such as cell debris and protein contaminant aggregations, were eluted from the C‐CP tip, as shown in Figure 2B. Also present are many globules of salt due to the presence of the 1‐M ammonium sulfate in the protein elution buffer. Interestingly, in Figure 2C, the second protein elution step results in a much cleaner image in terms of spurious debris, along with the release of a collection of small (∼30 nm), vesicle‐like species. Based on a TEM analysis alone, no comments can be made on the actual identity. This population of vesicles eluted during the protein elution step could consist of lipoproteins or so‐called exomeres, given their presence in cell culture conditions and lesser hydrophobicity in comparison to EVs. Based on previous MS proteomic and fluorescence studies [34, 56], this elution fraction is likely enriched in lipoproteins. The TEM micrograph of the targeted EV elution fraction is presented in Figure 2D, where vesicles of 30–298 nm (144‐nm average diameter) are observed. Many of the vesicles visualized in Figure 2D contain the characteristic cup or dimpled shape, with few matrix contaminants shown and the absence of large protein aggregates and the 20–40‐nm fraction of the vesicles. The presented TEM micrographs verify the ability to obtain structurally preserved EVs of the correct size from the HEK293 CCM using the C‐CP spin‐down tip method with the 1% Tween elution buffer.

### 3.3 Quantification of recovered EVs as a function of culture time and EV yield per cell

**FIGURE 3:**
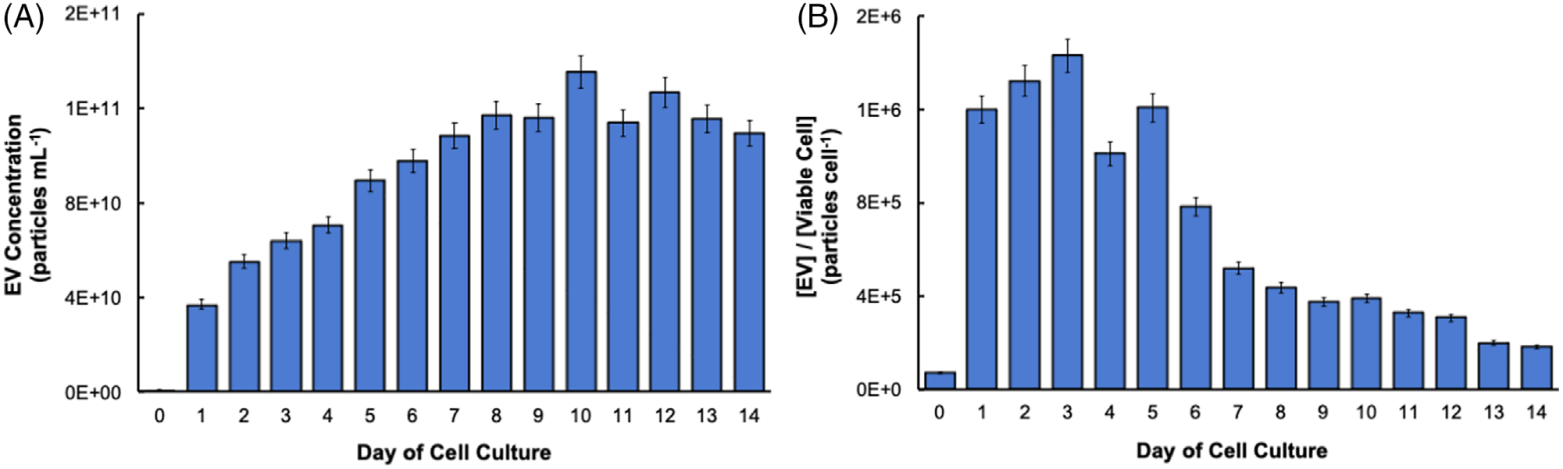
(A) Concentration of extracellular vesicles (EVs) collected from each cell culture milieu (CCM) aliquot using the capillary‐channeled polymer (C‐CP) tip isolation method and (B) concentration of EVs released per viable cell. Quantification performed using the method of standard addition via absorbance detection at 203 nm

The C‐CP tip method allows for the isolation of highly concentrated EV samples in a quantitative and reproducible manner, using minute (100 µl) sample volumes [35–37, 40]. These qualities are ideal in the case of small population (analytical) sampling of large‐scale cell culture conditions to monitor the health of the cell line based on EV production. The cell milieu collections from each time point were processed by the C‐CP tip EV isolation method, and the eluted EVs were quantified using the method of standard addition with absorbance detection at 203 nm. As shown in Figure 3A, the EVs isolated from the initial seeding aliquot of the cells into the new media and suspension culture flask yielded an EV concentration of 8.9 × 10^8^ particles ml^−1^. In only 24 h, a ∼40‐fold increase in EV concentration was realized (3.7 × 10^10^ particles ml^−1^). Further, with each day of cell culture, there was an increase in EV secretion until day 7, where the secreted EV concentration plateaus (1.1–1.4 × 10^11^ EVs ml^−1^). This is reflective of typical healthy cell growth and proliferation on days 0–7 of cell culture, where likely beyond the day‐7 time point, the cells become overpopulated, and the cell multiplication begins to decrease as the cell culture nutrients are depleted, and cell waste by‐products, such as lactate, begin to inhibit cell growth [51, 57, 58]. This is further confirmed by the total number and percentage of viable cells shown in Figure 1, where beyond day 7 of culture, the viability of the cells decreases below 80%, remaining on the level of ∼70%, and the number of recovered reaches a plateau. Important across this set of EV number determinations and the subsequent methods of characterization is the very high level of measurement precision, wherein triplicate determinations fall below 10% RSD, and impressive value in comparison to more traditional EV isolation methods [40]. Additionally, the relatively high values of ∼10^11^ particles ml^−1^ in the 100‐µl aliquots is easily accommodated on the 1‐cm C‐CP fiber tips [35].

It is well known that there is a practical difference between the *viability* of cells in a given culture and their *productivity* toward an end product [13, 21, 59]. Not only this concept would certainly be of relevance in the production of EVs as vectors but also may allow for EV production to provide insights into cellular processes. In Figure 3B, the concentration of recovered EVs is presented with respect to the viable cell concentration on each day of the cell culture process. After the isolation of EVs from the initially seeded cells, 7 × 10^4^ EVs per viable HEK293 cell were collected, which is reasonable as the viable cells were just released into the new media‐containing suspension flasks, and a minuscule amount of time was allowed to pass—lowering the probability for the occurrence of cellular communication processes (therefore, EV release). Still, the initially collected EVs were likely released into the cell culture flask in response to the cell seeding process, a physical stressor for the seeded cells [48, 60–62]. After the first 24 h of incubation, a 17‐fold increase in the concentration of EVs secreted per cell is observed (1.2 × 10^6^ EVs per cell). This high level of EV secretion per cell is observed on days 1–5, with a dramatic 50% decrease in EV productivity observed on day 6, followed by a steady decrease up to 14 days of culture. The drop in EV productivity corresponds with the onset of lower cell viability (Figure 1), though it has been suggested that as culture media components become depleted with time, they continuously become nutrient‐deprived and begin to prioritize cargo preservation, causing the EV output to decrease [57, 58]. Alternatively, the initial increase in EV yield may reflect an accelerated expression rate during the cell number growth phase, slowing as that process reaches a steady state. Though none of the identified works have monitored EV release during the production of therapeutic vectors/products, it would be interesting to assess potential relationships between the productivity of mAb‐ or viral vector‐producing cell lines and EV release characteristics [63, 64, 65, 66].

### 3.4 CD9 and CD81 expression of HEK293T/17 EVs

Despite the absence of a discrete EV biomarker to verify the bioactivity and quantity of EVs, antibodies to the CD9 and CD81 tetraspanin proteins are commonly employed during immunoassays to verify the identity of exosomes and other EVs based on the presence of the proteins on the vesicular surfaces [1, 67–69]. It is important to note that some individual tetraspanin proteins (including CD9 and CD81) are also expressed in the plasma membranes and endosomal/lysosomal compartments of cells; therefore, these (free) proteins could be present to some extent in CCM samples [67]. Regardless of the various origins of the proteins, antibodies to CD9 and CD81 have been used in numerous immunoassays to verify the presence of EVs [70, 71]. An indirect ELISA approach was used here to identify the C‐CP tip‐recovered EVs based on the tetraspanin proteins in the collections of CCM from each time point. Because of the heterogeneity of EV protein expression, even for EVs of the same origin and exposed to identical conditions, one cannot assume that the tetraspanin protein expression is directly correlated with the absolute concentration of EVs [18, 26]. That said, the absolute identification of tetraspanin proteins on the surface of the EVs is a confirmation of their identity and is suggestive of their retention of surface protein activity.

**FIGURE 4:**
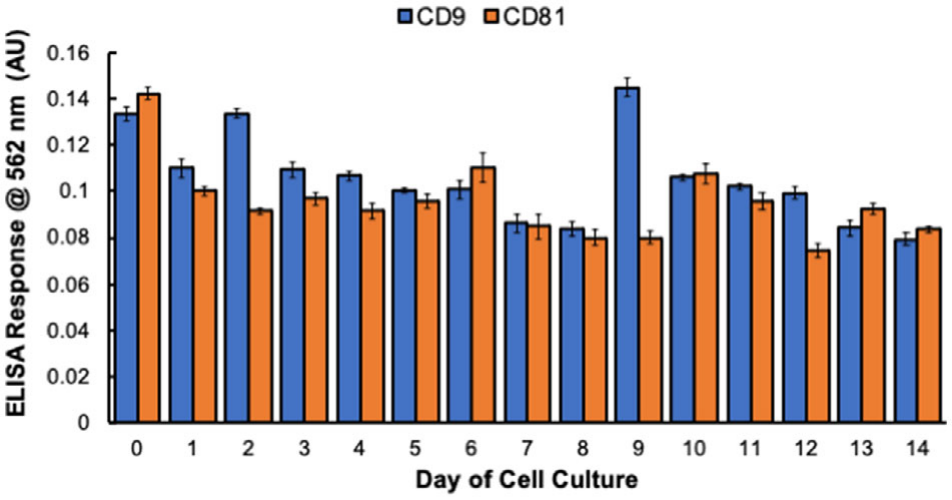
CD9 and CD81 tetraspanin protein responses of capillary‐channeled polymer (C‐CP) tip isolated extracellular vesicle (EV) recoveries from each time point, determined using an indirect enzyme‐linked immunosorbent assay (ELISA). All samples were applied in triplicate with the average of the triplicate measurements minus the average response of the blank is presented.

The responses to the ELISA assays for CD9 and CD81 over the course of the culture program are presented in Figure 4. As can be seen, the expression of the two tetraspanins remains relatively constant across the incubation period, with the absolute responses for the two proteins being fairly equivalent. This is a fortuitous situation and cannot be interpreted as meaning that the vesicular surface concentrations for the two species is actually the same. Across the entire suite of analyses, the triplicate isolation procedures (as well as the assay steps) are indeed very reproducible; a consistent feature of the C‐CP tip isolation methodology. It is interesting to note that there are specific sampling days (e.g., day 9) where the production of CD9 is clearly enhanced. It is beyond the scope of this effort to interpret the underlying reasons. As a final note, it is interesting that the ELISA responses remain consistent even though the raw number of EVs changes in the course of the culture cycle. This might suggest some sort of bias in the assay, but these samplings were all run in parallel with suitable controls/blanks. It may be that the production of tetraspanins themselves may be an indication of the health of the cell line. The ability to rapidly and repeatably obtain this information will provide researchers with the opportunities to investigate these relationships.

### 3.5 Size of recovered EVs via MALS

**FIGURE 5:**
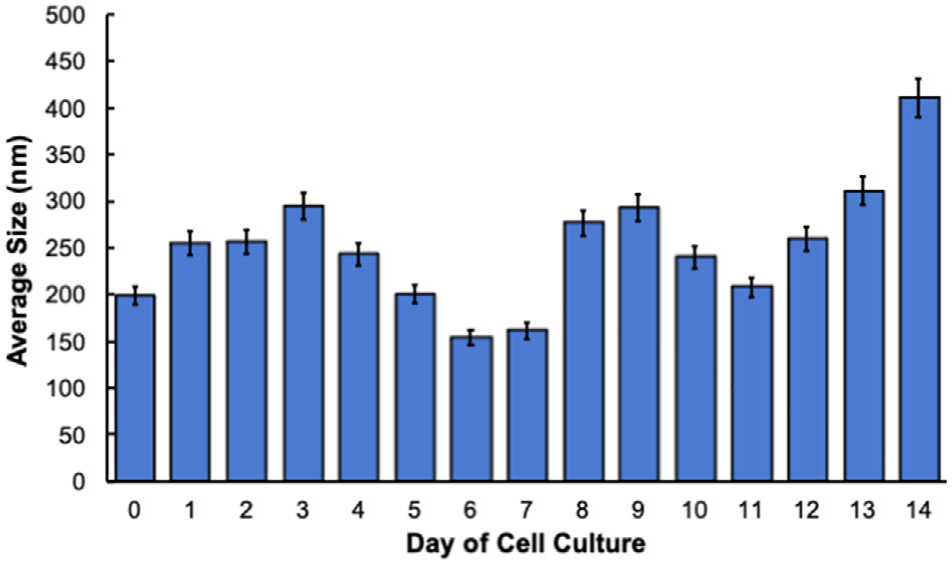
Size determination of the extracellular vesicles (EVs) recovered using the capillary‐channeled polymer (C‐CP) tip isolation method on each day of cell culture, performed using the Wyatt Dawn multi‐angle light scattering (MALS) instrument. Presented is the average size of the EVs resulting from three consecutive 60‐s runs.

Most commonly in EV research, nanoparticle tracking analysis (NTA) methods are used for EV size determinations [72, 73]. Previous use of the NTA instrument for evaluating EV size in this laboratory and others has raised concerns about the accuracy and precision of determinations due to significant inconsistencies in standard analyses [19, 36, 40, 74, 75]. The NTA methodology is susceptible to many different forms of interference, with the results having a tendency to be very operator dependent. To potentially circumvent the limitations of the NTA approach, researchers have previously employed MALS instruments for EV size determinations [14, 76, 77]. MALS size determination was used here to confirm that the EVs collected from the CCM samples had sizes that were within expected ranges. More importantly, the use of MALS in combination with the C‐CP tip isolation method was hoped to yield far higher levels of precision than previously obtained using NTA. Finally, as a flow‐through detection method, it is anticipated that the approach can be integrated into C‐CP fiber column‐based separations that are performed on standard HPLC instruments [32, 34, 39, 78]. The average diameters of the EVs isolated from the cellular milieu samples are shown in Figure 5. The eluted EVs presented average diameters of 145–411 nm across the CCM sample collections across the incubation period, with an average diameter of 249 nm overall. In comparison to previously obtained populations of EVs collected using the C‐CP tip, the average diameter of the vesicles is 50–100‐nm larger than those obtained from human biofluids using the ACN or glycerol solvent systems and NTA determinations [35, 40]. The significant difference in EV size could potentially be due to the use of the Tween‐20 EV elution buffer or could be a basic characteristic of the suspension‐adapted HEK293 source. Nonetheless, the relative precision of the EV size determinations using the MALS instrument is excellent, with less than 7% RSD across triplicate measurements of EV size. Given the high level of precision, the clear systemic (cyclic) variations in EV sizes may be of biological significance and worthy of further investigation. The assessment of the various C‐CP tip elution solvents in parallel isolations of EVs from identical sources is undoubtedly warranted for future experimentation, as is a direct comparison of determination methods, including NTA, MALS, and dynamic light scattering.

### 3.6 Protein concentration of cell culture milieu and purity assessment of recovered EVs

**FIGURE 6:**
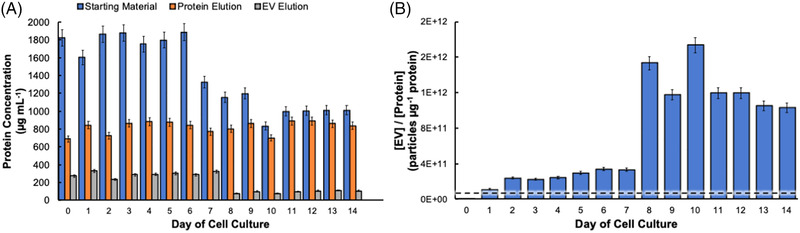
(A) Concentration of protein in HEK293 sample stocks and extracellular vesicle (EV) eluates from the capillary‐channeled polymer (C‐CP) tip at each step in the isolation, determined using a Bradford assay. (B) EV purity based on the ratio of the number of EVs to the mass of protein in the isolates. All samples applied in triplicate and the average of the triplicate measurements minus the average response of the blank. Dashed line indicates target purity level of 3 × 10^10^ EV µg^−1^ protein.

Bradford assays are commonly utilized to determine the total amino acid/protein content of diverse biological samples [47]. Here, the Bradford assay was used to investigate the purity of the EVs recovered from the HEK293 cell culture collections based on the removal of host cell proteins. To clarify, the response to the Bradford assay reflects the total proteinaceous material present in a sample. Therefore, even in the case of “pure” EVs, a positive yet lower Bradford response results due to the interaction between the Bradford reagent and the externally exposed EV‐associated proteins and amino acid residues. Figure 6 shows the Bradford assay‐determined protein concentrations for the raw CCM supernatants and the eluates of the subsequent C‐CP tip processing steps; that is, the “protein” and “exosome” fractions. As shown in Figure 6A, the CCM supernatant collections from days 0–6 of cell culture contained a consistent level of ∼1800 µg ml^−1^ of protein. Then, on days 7–14 of culture, the protein concentration drops to the level of ∼1000 µg ml^−1^. Efforts by Martinez‐Monge et al. have suggested that with increasing cell culture time comes inhibited HEK293 cell growth due to the presence of harmful cell waste by‐products, which causes a decrease in protein expression efficiency [51]. Indeed, this change in total protein content appears to correspond to the point where the percentage of viable cells drops significantly (Figure 1). This drop in “protein” content in the supernatant is not seen at all in the values derived in the first fiber tip wash step. Herein, the complementary aspects of the determinations may provide significant insights. Many previous efforts using the C‐CP fiber phases have shown that small polar/ionic molecules are not retained on the fibers, as such the first‐wash eluates should not contain proteins, but amino acids. The impact here is that the drop in “total protein” content in the supernatant observed after day 6 may be more reflective of decreased amino acid content in the CCM, not proteins *per se*. Processing the CCM samples using the complete C‐CP tip protocol reduces the apparent protein concentrations of each sampling by 76%–95% for the “EV” fractions. In each case, a high level of precision is seen following the Bradford assay, with the variability of each triplicate determination being <5% RSD. It is noteworthy that the time response of the protein concentrations in the final eluate parallels those of the supernatant samples, reflecting a very consistent level of overall purification efficiency.

Ultimately, the goal of any EV extraction protocol, be the end application fundamental research, clinical diagnostics, or vector production, is the isolation of the vesicles to the exclusion of the diversity of CCM constituents, most specifically proteins. The most common metric used to assess the purity of EV isolates is the fraction relationship between the number of EVs per mass of protein in the isolate, with >3 × 10^10^ EVs µg^−1^ of protein considered to be “high purity” [47]. As recently demonstrated for the case of human urine‐derived EVs, this is one of the metrics where the C‐CP fiber tip method excels in comparison to other methods [40]. The relationship between EV and the protein concentration (i.e., purity) is depicted across the culture cycle in Figure 6B. Highly pure EV collections were obtained on days 1–14 of cell culture, whereas those EVs collected on day 0 are considered “impure” simply because of the low concentration of EVs obtained at the initial cell seeding. In every other case, the determined values exceed the purity target of 3 × 10^10^ EV µg^−1^ protein (designated by dashed line) by a full order of magnitude. Indeed, in the case of the low‐viability cell conditions (beyond day 7), the values exceed the target by almost two orders of magnitude. In those latter data, the variability observed (<10% RSD) is due to the low protein values via the Bradford assay. Overall, these findings are in accordance with previous demonstrations of EV isolations using the C‐CP tip, where the purity of the tip‐recovered vesicles well exceeds the purity of vesicles processed using competitive ultracentrifugation or polymeric precipitation methods for EV isolation [35, 40], on shorter time scales, low sample volumes, and lower capital costs.

## 4 CONCLUDING REMARKS

There is a pressing need for methods to rapidly isolate, purify, and characterize EVs across very different size scales and matrices. The needs touch areas of fundamental biochemical research, clinical diagnostics, and vector production. In all, the C‐CP tip isolation method employing the Tween solvent is able to produce highly concentrated, pure, structurally preserved collections of EVs in a manner that is relevant in the scales of time, cost, and practicality, for fundamental research and clinical applications, with downstream applications of cell culture–sourced EVs holding promise using the fiber column format. The C‐CP tip isolation method was applied here to the isolation of HEK293‐derived EVs, suggested as a vector for the delivery of biotherapeutics. The C‐CP tip method provides rapid isolation, which provides high‐purity materials for subsequent characterization via a multitude of analytical methods. Initial characterization included the evolution of the purity of the materials via TEM imaging. The absorbance‐based quantification approach allows the tracking of EV release during the course of the cell culture process, where rapid processing of small aliquots (100 µl) of CCM would be advantageous for process monitoring. The C‐CP tip isolation method provided bioactive EVs of up to 1.4 × 10^11^ EVs ml^−1^ concentration, as verified via ELISA determinations. Ultimately, the purity of the derived EVs exceeded the target metrics in all relevant cases, by greater than one order of magnitude, with up to 95% removal of contaminant host cell proteins at various time points in cell culture. As presented, the method demonstrated here should allow researchers across diverse fields to gain greater fundamental information as to the roles of EVs in cell culture processes or as means of process monitoring. That said, extension to higher volume, preparative applications is a promising avenue as well.

## AUTHOR CONTRIBUTION

Kaylan K. Jackson: Methodology, data curation, visualization, writing—original draft preparation; R. Kenneth Marcus: Conceptualization, supervision, writing—reviewing and editing.

## CONFLICT OF INTEREST

The authors have declared no conflict of interest.

## Data Availability

The data that support the findings of this study are available on request from the corresponding author. The data are not publicly available due to privacy or ethical restrictions.
